# Comparisons of the Relationships Between Multiple Lipid Indices and Diabetic Kidney Disease in Patients With Type 2 Diabetes: A Cross-Sectional Study

**DOI:** 10.3389/fendo.2022.888599

**Published:** 2022-07-06

**Authors:** Chun-feng Lu, Wang-shu Liu, Zhen-hua Chen, Ling-yan Hua, Xue-qin Wang, Hai-yan Huang

**Affiliations:** ^1^ Department of Endocrinology, Affiliated Hospital 2 of Nantong University and First People’s Hospital of Nantong City, Nantong, China; ^2^ Department of Neurosurgery, Affiliated Hospital 2 of Nantong University and First People’s Hospital of Nantong City, Nantong, China; ^3^ Department of Ophthalmology, Affiliated Hospital 2 of Nantong University and First People’s Hospital of Nantong City, Nantong, China

**Keywords:** type 2 diabetes, diabetic kidney disease, lipid indices, low-density lipoprotein cholesterol/apolipoprotein B ratio, small dense low-density lipoprotein cholesterol

## Abstract

**Background:**

Dyslipidemia is a well-recognized risk factor for diabetic kidney disease (DKD) in patients with type 2 diabetes (T2D). Growing evidences have shown that compared with the traditional lipid parameters, some lipid ratios may provide additional information of lipid metabolism. Thus, the present study aimed to investigate which lipid index was most related to DKD.

**Methods:**

This study was a cross-sectional study that enrolled patients with T2D from January 2021 to October 2021. Each participant was screened for DKD, and the diagnostic criterion for DKD is estimated glomerular filtration rate (eGFR) < 60 ml/min/1.73 m^2^ or urinary albumin-to-creatinine ratio (UACR) ≥ 30 mg/g for 3 months. Fasting blood was collected to determine lipid profiles by an automatic biochemical analyzer, and lipid ratios were calculated based on corresponding lipid parameters. Spearman’s correlation analyses were conducted to assess the correlations between lipid indices and kidney injury indices, and binary logistic regression analyses were conducted to explore the relationship between lipid indices and the risk of DKD.

**Results:**

A total of 936 patients with T2D were enrolled in the study, 144 (15.38%) of whom had DKD. The LDL-C/Apo B ratios were positively correlated with eGFR (*r =* 0.146, *p <* 0.05) and inversely correlated to cystatin C and UACR (*r =* -0.237 and -0.120, both *p <* 0.001). Multiple logistic regression demonstrated that even after adjusting for other clinical covariates, the LDL-C/Apo B ratios were negatively related to DKD, and the odds ratio (95% confidence interval) was 0.481 (0.275–0.843). Furthermore, subgroup analyses revealed that compared with patients with normal lipid profiles and a high LDL-C/Apo B ratio, the odds ratio of DKD in patients with normal lipid metabolism and a low LDL-C/Apo B ratio was 2.205 (1.136-4.280) after adjusting for other clinical covariates.

**Conclusion:**

In patients with T2D, the LDL-c/Apo B ratio was most closely associated with DKD among various lipid indices, and a lower LDL-C/Apo B ratio was associated with increased risks of DKD among patients with T2D.

## Introduction

As one of the major microvascular complications of type 2 diabetes (T2D), diabetic kidney disease (DKD) affects about 20% of patients with T2D (1). The presence of DKD not only is susceptible to progress to end-stage renal disease requiring dialysis (2), but also significantly increases the risk of cardiovascular disease (CVD) (3), thus creating a great impaction on patients with T2D. An integrated approach should be taken for the prevention and control of DKD for the fact that DKD is a multifactorial disease. Among them, lipid metabolism disorder exerts key roles on the pathogenesis of DKD and functions as an important target for DKD prevention and treatment. Dyslipidemia in patients with T2D is typically characterized with high levels of triglyceride (TG) and low-density lipoprotein cholesterol (LDL-C), and low level of high-density lipoprotein cholesterol (HDL-C) (4). Statins, the mainstay of current lipid-lowering drugs, reduce plasma levels of LDL-C and TG through inhibiting cholesterol synthase (5). However, although statins are recommended by all available guidelines and can reduce proteinuria and all-cause mortality, they fail to delay the progression of end-stage renal disease (6). Therefore, it is plausible that it is not fully to reflect the risk of DKD based on the traditional lipid indices, and it is desirable to search for new lipid-lowering therapeutic targets.

In recent years, growing evidences have shown that compared with the traditional lipid indices, apolipoprotein A1 (Apo A1), apolipoprotein B (Apo B) and lipid ratios may provide additional information of lipid metabolism. Apo A1 and Apo B are structural components of HDL-C and atherosclerotic lipoprotein respectively (7), thus the Apo B/Apo A1 ratio is a surrogate maker of the cholesterol balance between atherogenic and antiatherogenic lipoprotein particles. Multiple studies have demonstrated that the Apo B/Apo A1 ratio is closely associated with cardiac vulnerable plaques (8), and in-stent restenosis (9). Zhao et al. also revealed that a high level of Apo B/Apo A1 ratio can predict the progression of DKD (10). Moreover, the TG/HDL-C ratio, HDL-C/Apo A1 ratio and LDL-C/Apo B ratio have drawn extensive attention. TG/HDL-C ratio is identified as an atherosclerosis index, and a longitudinal follow-up study showed that the TG/HDL-C ratio was a predictor of microvascular complications in patients with T2D (11). Other research has shown that the HDL-C/Apo A1 ratio and LDL-C/Apo B ratio are closely related to the deterioration of glycemia and the onset of T2D (12). In a word, these lipid indices may be more conducive to DKD risk stratification in patients with T2D, but few studies have focused on comparing the relationships between these parameters and DKD in patients with T2D. Therefore, the present study was designed to explore which lipid index is optimal-related to DKD in patients with T2D.

## Methods

### Study Design and Participants

In this observational cross-sectional study, patients diagnosed with T2D according to the statement of the American Diabetes Association and screened for DKD were enrolled from the inpatient department of the Second Affiliated Hospital of Nantong University from January 2021 to October 2021 (13). The following cases were excluded: type 1 diabetes, secondary diabetes, previous and current malignant tumors, chronic hepatitis and heart failure, acute diabetic complications and other kidney diseases and urinary tract infection. Finally, 936 patients with T2D were included in the present study. After full understanding of the present study protocol, each subject signed written informed consent. The study was in accordance with the Declaration of Helsinki and approved by the medical research ethics committee of the Second Affiliated Hospital of Nantong University.

### Basic Data Collection

At enrollment, all subjects completed a questionnaire with the assistance of experienced physicians to collect the demographic data, lifestyle, medication history and diagnosis history of diseases. Body mass index (BMI) was calculated as the weight/height squared. Blood pressure was measured by a standard mercury sphygmomanometer, and the average of three recordings was recorded.

### Laboratory Examination and Calculation

After enrollment, fasting blood samples and fresh first-void morning urine samples were taken for measurement of laboratory parameters, urinary albumin and urinary creatinine, respectively. Lipid profiles and urinary creatinine were measured by an automated biochemical analyzer (Model 7600, Hitachi), and lipid ratios were calculated based on corresponding lipid parameters. Urinary albumin level was evaluated by the immunoturbidimetry method (Immage 800, Beckman Coulter). The algorithm of urinary albumin creatinine ratio (UACR) was the ratio of urinary albumin to urinary creatinine. Estimated glomerular filtration rate (eGFR) was calculated based on the CKD-EPI creatinine-cystatin C equation (2012) (14). If a patient with T2D has an eGFR < 60 ml/min/1.73 m^2^ or a UACR ≥ 30 mg/g lasted for more than 3 months, a DKD diagnosis can be made (15).

### Grouping Criteria

To explore the relationship between low LDL-C/Apo B ratio and the risk of DKD in T2D patients with normal lipid profiles, the total population was divided into A, B, C and D four groups according to lipid profiles and LDL-C/Apo B ratio. In our laboratory, the reference ranges of lipid metabolism parameters TG, TC, HDL-C and LDL-C were respectively 0.2 - 2.0 mmol/L, 2.9 – 6.0 mmol/L, 1.1 – 1.7 mmol/L and 1.55 – 3.35 mmol/L. Therefore, dyslipidemia was defined as any lipid parameter above the normal ranges described above. Then, a low or a high LDL-C/Apo B ratio was defined according to the median of LDL-C/Apo B ratio (2.8804). Ultimately, group A, B, C and D respectively represented normal lipid profile with a high LDL-C/Apo B ratio, normal lipid profile with a low LDL-C/Apo B ratio, abnormal lipid profile with a high LDL-C/Apo B ratio and abnormal lipid profile with a low LDL-C/Apo B ratio.

### Statistical Analysis

The clinical characteristics of the participants grouped by DKD status were described by mean ± SD, median (25 and 75% interquartile) and frequencies (percentages) for the normally and skewed distributed continuous variables and the categorical variables, respectively. We adopted Student’s t-test to compare differences in normally distributed data, the Mann–Whitney test to compare differences in skewed distributed data and the chi-square test to compare categorical data. Spearman’s bivariate correlation analyses were conducted to analyze the correlations of multiple lipid indices with DKD indicators. As the LDL-C/Apo B ratio was the only parameter related to DKD, multivariate logistic regression analyses were performed to investigate the impact of the LDL-C/Apo B ratio on DKD. Furthermore, the differences in the proportion and odds ratio (OR) were compared among group A, B, C and D by the chi-square test and multivariate logistic regression analyses. All analyses were performed using SPSS statistical software 18.0 (IBM SPSS Inc., USA). A value of *p <* 0.05 was defined as statistical significance.

## Results

### Clinical Characteristics of the Study Participants

Among the recruited 936 T2D patients, patients combined with DKD accounted for 15.38%. As shown in **Table 1**, T2D patients with DKD had older ages, longer diabetic durations, higher systolic blood pressures, a higher prevalence of hypertension, higher prevalence of insulin, and statins users, higher blood urea nitrogen (BUN) levels, creatinine (Cr) levels, uric acid (UA) levels, cystatin C levels, UACR levels, lower eGFRs, and lower LDL-C/Apo B ratios (all *p <* 0.05) than T2D patients without DKD. There were no differences in proportion of males, BMI, diastolic blood pressures, uses of other antidiabetic treatments other than insulin, HbA1c levels and lipid indices other than LDL-C/Apo B ratio between patients with and without DKD (p > 0.05).

**Table 1:** Clinical characteristics of the study participants.

Variables		T2D		*p* value
	Total	Without DKD	With DKD	
*n*	936	792	144	
Age (years)	57.65 ± 13.60	55.59 ± 13.20	68.98 ± 9.65	<0.001
Male, *n* (%)	533 (56.9)	458 (57.8)	75 (52.1)	0.202
Diabetic duration (years)	6.0 (1.0-10.0)	5.0 (1.0-10.0)	10.0 (5.8-20.0)	<0.001
Smoking history, *n* (%)	85 (9.1)	68 (8.6)	17 (11.8)	0.210
BMI (kg/m^2^)	25.62 ± 3.94	25.63 ± 4.04	25.55 ± 3.36	0.842
Hypertension, *n* (%)	345 (36.9)	260 (32.8)	85 (59.0)	<0.001
SBP (mmHg)	134 (123-147)	132 (123-145)	142 (126-157)	<0.001
DBP (mmHg)	81.37 ± 11.04	81.43 ± 10.50	81.08 ± 13.69	0.726
Antidiabetic treatments				
Insulin treatment, *n* (%)	242 (25.9)	182 (23.0)	60 (41.7)	0.001
Metformin, *n* (%)	415 (44.3)	350 (44.2)	65 (45.1)	0.856
Acarbose, *n* (%)	82 (8.8)	68 (8.6)	14 (9.7)	0.632
Insulin-secretagogues, *n* (%)	284 (30.3)	239 (30.2)	45 (31.3)	0.844
Insulin-sensitisers, *n* (%)	77 (8.2)	64 (8.1)	13 (9.0)	0.741
DPP-4 inhibitors, *n* (%)	44 (4.7)	35 (4.4)	9 (6.3)	0.389
SGLT-2 inhibitors, *n* (%)	66 (7.1)	52 (6.6)	14 (9.7)	0.213
Antihypertensive treatments				
CCB, *n* (%)	230 (24.6)	169 (21.4)	61 (42.4)	<0.001
ARB, *n* (%)	188 (20.1)	145 (18.3)	43 (29.9)	0.002
β-blockers, *n* (%)	45 (4.8)	26 (3.3)	19 (13.2)	<0.001
Diuretics, *n* (%)	76 (8.1)	54 (6.8)	22 (15.3)	0.001
Statins medications, *n* (%)	58 (6.2)	35 (4.4)	23 (16.0)	<0.001
HbA1c (%)	9.32 ± 2.16	9.35 ± 2.14	9.14 ± 2.27	0.284
BUN (mmol/L)	5.23 (4.32-6.62)	5.11 (4.20-6.26)	7.13 (5.32-9.16)	<0.001
Cr (umol/L)	56.0 (47.0-67.0)	54.0 (46.0-63.0)	86.0 (68.0-116.0)	<0.001
Serum UA (umol/L)	301.0 (241.0-375.0)	290.5 (232.3-354.8)	380.0 (313.0-472.0)	<0.001
Cystatin C (mg/L)	0.83 (0.67-1.02)	0.78 (0.64-0.93)	1.33 (1.15-1.65)	<0.001
eGFR (ml/min/1.73m^2^)	102.93 ± 29.22	110.58 ± 23.22	58.60 ± 19.31	<0.001
UACR (mg/g)	16.25 (8.10-47.65)	13.60 (7.70-31.98)	141.45 (24.15-897.55)	<0.001
TG (mmol/L)	1.64 (1.07-2.67)	1.62 (1.06-2.59)	1.79 (1.14-2.83)	0.357
TC (mmol/L)	4.34 (3.72-5.01)	4.36 (3.74-5.03)	4.27 (3.57-4.96)	0.121
HDL-C (mmol/L)	1.12 (0.95-1.31)	1.12 (0.96-1.31)	1.12 (0.92-1.30)	0.360
LDL-C (mmol/L)	2.73 ± 0.88	2.75 ± 0.86	2.61 ± 1.00	0.071
Apo A1 (mmol/L)	1.06 (0.97-1.19)	1.07 (0.97-1.20)	1.05 (0.94-1.19)	0.115
Apo B (mmol/L)	0.94 (0.75-1.09)	0.94 (0.75-1.09)	0.93 (0.77-1.07)	0.885
TG/HDL-C (mmol/mmol)	1.44 (0.88-2.72)	1.43 (0.86-2.72)	1.59 (0.94-2.82)	0.239
LDL-C/Apo B (mmol/mmol)	2.88 (2.56-3.3)	2.91 (2.59-3.34)	2.74 (2.38-3.08)	<0.001
HDL-C/Apo A1 (mmol/mmol)	1.03 (0.93-1.13)	1.03 (0.93-1.13)	1.02 (0.92-1.12)	0.909
Apo B/Apo A1 (mmol/mmol)	0.88 ± 0.27	0.87 ± 0.27	0.90 ± 0.29	0.204

Normally distributed values in the table are given as the mean ± SD, skewed distributed values are given as the median (25 and 75% interquartiles), and categorical variables are given as frequency (percentage).

T2D, type 2 diabetes; DKD, diabetic kidney disease; BMI, body mass index; SBP/DBP, systolic/diastolic blood pressure; DPP-4 inhibitors, dipeptidyl peptidase-4 inhibitors; SGLT-2 inhibitors, sodium-glucose co-transporter-2 inhibitors; CCB, calcium channel blockers; ARB, angiotensin receptor blockers; HbA1c, glycosylated hemoglobin A1c; BUN, blood urea nitrogen; Cr, creatinine; Serum UA, serum uric acid; eGFR, estimated glomerular filtration rate; UACR, urine albumin/creatinine ratio; TG, triglycerides; TC, total cholesterol; HDL-C, high-density lipoprotein cholesterol; LDL-C, low-density lipoprotein cholesterol; Apo A1, apolipoprotein A1; Apo B, apolipoprotein B.

### Relationships Between Lipid Indices and Kidney Damage Indices in Patients With T2D

**Table 2** shows that the LDL-C/Apo B ratio was positively correlated with eGFR (*r =* 0.146, *p <* 0.05), and negatively correlated with cystatin C levels and UACR (*r =* -0.237 and -0.120, both *p <* 0.05). Cystatin C levels were significantly correlated with HDL-C levels, Apo B levels, HDL-C/Apo A1 ratios and Apo B/Apo A1 ratios (*r =* -0.102, 0.074, 0.084, -0.129 and 0.116, respectively, all *p <* 0.05). UACR were positively correlated with TG levels and TG/HDL-C ratios (*r =* 0.127 and 0.123, both *p <* 0.05). However, there were no significant correlations between kidney damage indices and total cholesterol (TC) levels and Apo A1 levels (all *p >* 0.05).

**Table 2:** Relationships between lipid indices and kidney damage indices in patients with T2D.

Lipid indices	Cystatin C		UACR		eGFR	
	*r*	*p* value	*r*	*p* value	*r*	*p* value
TG	-0.150	0.668	0.127	<0.001	0.046	0.190
TC	-0.130	0.210	0.034	0.311	0.119	0.245
HDL-C	-0.102	0.004	-0.027	0.420	0.026	0.454
LDL-C	0.074	0.028	-0.032	0.347	-0.077	0.329
Apo A1	-0.043	0.227	0.015	0.669	0.025	0.483
Apo B	0.084	0.017	0.050	0.143	-0.013	0.718
TG/HDL-C	0.017	0.621	0.123	<0.001	0.031	0.376
LDL-C/Apo B	-0.237	<0.001	-0.120	<0.001	0.146	<0.001
HDL-C/Apo A1	-0.129	<0.001	-0.045	0.191	0.051	0.152
Apo B/Apo A1	0.116	0.001	0.031	0.362	-0.039	0.267

r spearman’s correlation coefficient

T2D, type 2 diabetes; UACR, urine albumin/creatinine ratio; eGFR, estimated glomerular filtration rate; TG, triglycerides; TC, total cholesterol; HDL-C, high-density lipoprotein cholesterol; LDL-C, low-density lipoprotein cholesterol; Apo A1, apolipoprotein A1; Apo B, apolipoprotein B.

### Association of the LDL-C/Apo B Ratio With DKD in Patients With T2D

As the LDL-C/Apo B ratio was the only parameter correlated with all kidney damage indices, we thereby constructed multivariate logistic regression analyses to analyze the association between the LDL-C/Apo B ratio and DKD in patients with T2D. As illustrated in **Table 3**, DKD was significantly associated with the LDL-C/Apo B ratio [OR (95% CI), 0.560 (0.409-0.766)] in the basal unadjusted model 0. Even in the fully adjusted model 2, the LDL-C/Apo B ratio was still independently associated with DKD [OR (95% CI), 0.481 (0.275-0.843)].

**Table 3:** Multivariate logistic regression analysis to identify the association of LDL-C/Apo B ratio with DKD.

Models	*B*	SE	Wald	*p*	OR	95% CI
Model 0	-0.580	0.160	13.193	<0.001	0.560	0.409-0.766
Model 1	-0.620	0.205	9.124	0.003	0.538	0.360-0.804
Model 2	-0.731	0.286	6.546	0.011	0.481	0.275-0.843

Model 0: unadjusted model.

Model 1: adjusted for age, male, diabetic duration, smoking history, BMI, hypertension, SBP, DBP.

Model 2: additionally adjusted for HbA1c, antidiabetic treatments, antihypertensive treatments, statins medications.

### Proportion and ORs of DKD Based on Subgroup Analyses

**Figure 1:**
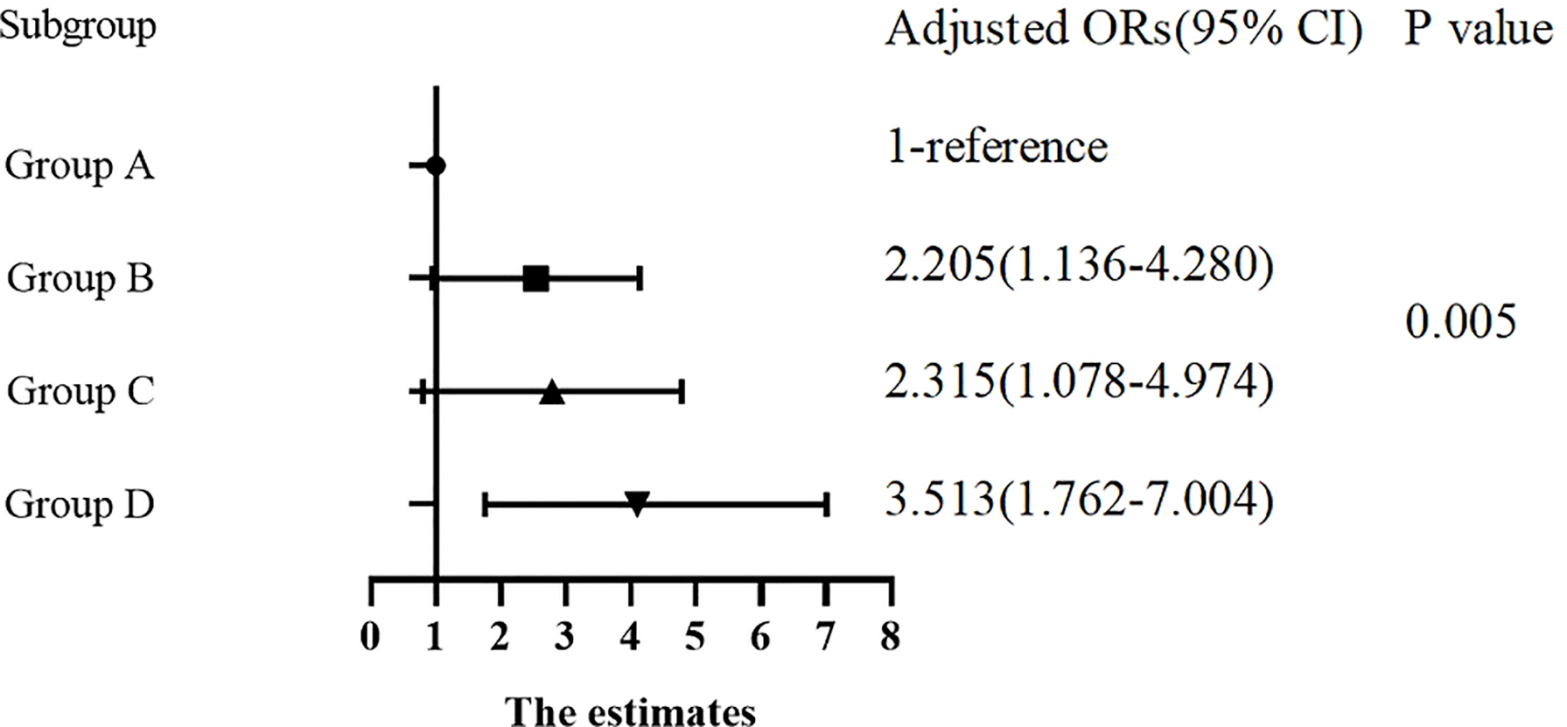
Forest plot visualizing adjusted ORs based on logistic regression analysis.

There were significant differences in the proportion of patients with DKD among Group A, B, C and D (8.7%, 21.3%, 12.8% and 19.7%, respectively, *p* for trend *<* 0.05). In **Table 4**, compared with Group A, the ORs of DKD in Group B, C and D were 2.850 (1.705-4.763), 1.557 (0.855-2.837) and 2.597 (1.538-4.385), respectively. After adjusting for other clinical factors, the ORs of DKD in Group B, C and D were 2.205 (1.136-4.280), 2.315 (1.078-4.974) and 3.513 (1.762-7.004) vs Group A, respectively (**Figure 1**).

**Table 4:** ORs (95% CIs) of DKD according to the four subgroups.

Variable	Group A	Group B	Group C	Group D	*P* value
LDL-C/Apo B ratio range	2.881-7.387	0.732-2.879	2.881-7.387	0.732-2.879	–
Number	289	240	179	228	–
DKD	25 (8.7)	51 (21.3)	23 (12.8)	45 (19.7)	<0.001
Model 0	1-reference	2.850 (1.705-4.763)	1.557 (0.855-2.837)	2.597 (1.538-4.385)	<0.001
Model 1	1-reference	2.190 (1.167-4.113)	2.235 (1.066-4.684)	3.743 (1.955-7.167)	0.001
Model 2	1-reference	2.205 (1.136-4.280)	2.315 (1.078-4.974)	3.513 (1.762-7.004)	0.005

Group A: normal lipid profile with a high LDL-C/Apo B ratio (> 2.880); Group B: normal lipid profile with a low LDL-C/Apo B ratio (< 2.880); Group C: abnormal lipid profile with a high LDL-C/Apo B ratio (> 2.880); Group D: abnormal lipid profile with a low LDL-C/Apo B ratio (< 2.880).

Model 0: unadjusted model.

Model 1: adjusted for age, male, diabetic duration, smoking history, BMI, hypertension, SBP, DBP.

Model 2: additionally adjusted for HbA1c, antidiabetic treatments, antihypertensive treatments, statins medications.

## Discussion

In the current study, we evaluated the associations between multiple lipid indices with the risk of DKD in patients with T2D. We found that patients with DKD had a lower level of LDL-C/Apo B ratio than patients without DKD. The LDL-C/Apo B ratio was significantly associated with eGFR, UACR and cystatin C. We also demonstrated that the LDL-C/Apo B ratio is independently related to the prevalence of DKD. Moreover, we revealed that even in patients with normal lipid profiles, a low level of LDL-C/Apo B ratio was relevant to an increased risk of DKD.

Hyperlipidemia is a well-recognized risk factor for DKD in patients with T2D. Hyperlipidemia can facilitate apoptosis of podocytes, a specialized kidney epithelial cell, thus damaging the integrity of the glomerular filtration barrier and eventually causing the onset of proteinuria (16). In addition to podocytes, hyperlipidemia can also promote glomerulosclerosis, interstitial fibrosis and accelerate the progression of proteinuria by affecting glomerular endothelial cells and mesangial cells and promoting the accumulation of collagen and fibronectin (17). Renal tubular injury is a vital component of DKD, and even precedes the occurrence of glomerular injury (18). Hyperlipidemia can cause renal tubular injury when combined with proteinuria by the mechanism that albumin can act as a carrier of fatty acids and promote the deposition of fatty acids in kidney (19). Additionally, ectopic deposition of lipids in kidney can induce local oxidative stress and inflammation *via* generating excess adipokines and activating a variety of signaling pathways (20). The present study demonstrated that the LDL-C/Apo B ratio was closely related to the prevalence of DKD in patients with T2D.

There have been many studies focused on investigating the relationships between lipid indices and DKD, but there are discrepancies among these studies. In a 2.9 years follow-up study, high levels of TG and low levels of HDL-C at baseline, but not levels of LDL-C could predict the decline of renal function (21). Slightly different from the above study, Retnakaran R et al. showed that serum TG and LDL-C levels were predictors of proteinuria in patients with T2D (22). In a longitudinal study involving a large Japanese population, the TG/HDL-C ratio was positively associated with the increase of proteinuria and the decline of eGFR (23). In contrast, no associations were observed between lipid indices and DKD or DKD markers in other studies (24–26). UACR, eGFR and cystatin C, respectively reflecting glomerular filtration barrier, glomerular filtration function and tubular function, all are important indicators of kidney function (27). In this study, we observed that both TG and TG/HDL-C ratio were only positively associated with UACR, while Apo B/Apo A1 ratio was only positively associated with cystatin C, which indicated that these lipid indices were insufficient to comprehensively assess renal injury in patients with T2D. These inconsistencies among these studies and our study may be attributed to differences in the included population, uses of statin. In this study, the LDL-C/Apo B ratio was significantly correlated with kidney injury indices, suggesting that LDL-C/Apo B ratio was most closely correlated with DKD among multiple lipid indices.

A review published in 2020 suggested that hyperlipidemia may promote atherosclerosis independent of LDL-C concentration, which may be partly attributed to the presence of small dense LDL-C (sd-LDL-C) (28). LDL-C is a group of apolipoproteins with greater heterogeneity, and LDL-C with small particle size and high density is named as sd-LDL-C (29). Methods such as nuclear magnetic resonance and hypervelocity centrifugation can be used to evaluate sd-LDL-C, but these methods are not suitable for clinical practice due to high costs and lengthy processes (30). The LDL-C/Apo B ratio widely used in clinical and research studies has been identified as a surrogate marker for sd-LDL-C, and the smaller the LDL-C/Apo B ratio, the greater the proportion of sd-LDL-C (31). The present study found that even in patients with normal lipid profiles, having a low LDL-C/Apo B ratio significantly increased the risk of DKD in patients with T2D.

Compared with other LDL-Cs, less sd-LDL-Cs binding to LDL-C receptors result in sd-LDL-C staying in the circulation longer (32), and sd-LDL-Cs tend to be oxidized and modified to form oxidized LDL (33). Oxidized LDL, a marker of endothelial dysfunction and oxidative stress, can be excessively uptaken by multiple types of kidney cells, thus promoting glomerulosclerosis and kidney fibrosis, eventually accelerating the process of DKD (34). In addition, macrophages can be attracted by oxidized LDL, chemotaxis and phagocytosis oxidized LDL to form foam cells. Subsequently, the accumulation of foam cells in renal arterioles aggravates local renal hemodynamic disorders (35). Sodium-glucose co-transporter-2 inhibitors are a novel class of hypoglycemic agents recommended for type 2 diabetic patients with DKD by a group of guidelines, and a clinical study showed that the use of SGLT2 inhibitors could significantly reduce the level of sd-LDL-C in patients with T2D (36). These results together suggested that a low LDL-C/Apo B ratio was an important risk factor and a potential target for DKD in patients with T2D.

This study had several limitations. First, as this study was a cross-sectional study, the causal relationship between the LDL-C/Apo B ratio and DKD could not be fully elucidated. Second, the LDL-C/Apo B ratio was a proxy for evaluating sd-LDL-C rather than the gold standard, but the large sample size of this study compensated for this deficiency. Third, the generalizability of this study was limited by that this study was based on a Chinese population. Therefore, longitudinal and intervention studies are needed to address the above limitations.

In summary, the LDL-C/Apo B ratio was closely associated with the risk of DKD, and a lower LDL-C/Apo B ratio might be a potent risk factor and therapeutic target for prevention and treatment of DKD in patients with T2D. Even in type 2 diabetic patients with normal lipid profile, the LDL-C/Apo B ratio should be routinely assessed.

## Data Availability Statement

The original contributions presented in the study are included in the article/supplementary material. Further inquiries can be directed to the corresponding authors.

## Ethics Statement

The studies involving human participants were reviewed and approved by The medical research ethics committee of Second Affiliated Hospital of Nantong University. The patients/participants provided their written informed consent to participate in this study.

## Author Contributions

C-FL and Z-HC participated in the design of the study, data collection, analysis of the data, and drafting of the manuscript. X-QW and H-YH conceived of the study, participated in its design and revised the manuscript. W-SL and L-YH participated in data collection. All authors read and approved the final manuscript.

## Funding

The study was supported by the Medical Research Project of Health Commission of Nantong (MB2020012, MA2020004) and the Science and Technology Support Program of Nantong (JCZ20127, JCZ21099, JC2020037).

## Conflict of Interest

The authors declare that the research was conducted in the absence of any commercial or financial relationships that could be construed as a potential conflict of interest.

## Publisher’s Note

All claims expressed in this article are solely those of the authors and do not necessarily represent those of their affiliated organizations, or those of the publisher, the editors and the reviewers. Any product that may be evaluated in this article, or claim that may be made by its manufacturer, is not guaranteed or endorsed by the publisher.

## References

[1] Domingueti CP Dusse LM Carvalho Md de Sousa LP Gomes KB Fernandes AP. Diabetes Mellitus: The Linkage Between Oxidative Stress, Inflammation, Hypercoagulability and Vascular Complications. J Diabetes Complications (2016) 30:738–45. doi: 10.1016/j.jdiacomp.2015.12.018 26781070

[2] Hosny SS Bekhet MM Hebah HA Mohamed NR. Urinary Neutrophil Gelatinase-Associated Lipocalin in Type 2 Diabetes: Relation to Nephropathy and Retinopathy. Diabetes Metab Syndr (2018) 12:1019–24. doi: 10.1016/j.dsx.2018.06.017 29960862

[3] Adler AI Stevens RJ Manley SE Bilous RW Cull CA Holman RR. Development and Progression of Nephropathy in Type 2 Diabetes: The United Kingdom Prospective Diabetes Study (UKPDS 64). Kidney Int (2003) 63:225–32. doi: 10.1046/j.1523-1755 12472787

[4] Taskinen MR. Diabetic Dyslipidaemia: From Basic Research to Clinical Practice. Diabetologia (2003) 46:733–49. doi: 10.1007/s00125-003-1111-y 12774165

[5] Bussolati B Deregibus MC Fonsato V Doublier S Spatola T Procida S. Statins Prevent Oxidized LDL-Induced Injury of Glomerular Podocytes by Activating the Phosphatidylinositol 3-Kinase/AKT-Signaling Pathway. J Am Soc Nephrol (2005) 16:1936–47. doi: 10.1681/ASN.2004080629 15843472

[6] Zhang Z Wu P Zhang J Wang S Zhang G. The Effect of Statins on Microalbuminuria, Proteinuria, Progression of Kidney Function, and All-Cause Mortality in Patients With non-End Stage Chronic Kidney Disease: A Meta-Analysis. Pharmacol Res (2016) 105:74–83. doi: 10.1016/j.phrs.2016.01.005 26776964

[7] Gao L Zhang Y Wang X Dong H. Association of Apolipoproteins A1 and B With Type 2 Diabetes and Fasting Blood Glucose: A Cross-Sectional Study. BMC Endocr Disord (2021) 21:59. doi: 10.1186/s12902-021-00726-5 33794863 PMC8017773

[8] Deng F Li D Lei L Yang Q Li Q Wang H. Association Between Apolipoprotein B/A1 Ratio and Coronary Plaque Vulnerability in Patients With Atherosclerotic Cardiovascular Disease: An Intravascular Optical Coherence Tomography Study. Cardiovasc Diabetol (2021) 20:188. doi: 10.1186/s12933-021-01381-9 34526013 PMC8442358

[9] Akutsu N Hori K Mizobuchi S Ogaku A Koyama Y Fujito H. Clinical Importance of the LDL-C/apolipoprotein B Ratio for Neointimal Formation After Everolimus-Eluting Stent Implantations. J Atheroscler Thromb (2022) 29:536–50. doi: 10.5551/jat.60954 PMC9090476 33746158

[10] Zhao WB Alberto P. Serum Apolipoprotein B/apolipoprotein A1 Ratio is Associated With the Progression of Diabetic Kidney Disease to Renal Replacement Therapy. Int Urol Nephrol (2020) 52:1923–8. doi: 10.1007/s11255-020-02550-7 32661625

[11] Zoppini G Negri C Stoico V Casati S Pichiri I Bonora E. Triglyceride-High-Density Lipoprotein Cholesterol is Associated With Microvascular Complications in Type 2 Diabetes Mellitus. Metabolism (2012) 61:22–9. doi: 10.1016/j.metabol.2011.05.004 21676418

[12] Fizelova M Miilunpohja M Kangas AJ Soininen P Kuusisto J Ala-Korpela M. Associations of Multiple Lipoprotein and Apolipoprotein Measures With Worsening of Glycemia and Incident Type 2 Diabetes in 6607 non-Diabetic Finnish Men. Atherosclerosis (2015) 240:272–7. doi: 10.1016/j.atherosclerosis.2015.03.034 25818853

[13] American Diabetes Association. Diagnosis and Classification of Diabetes Mellitus. Diabetes Care (2013) 36 Suppl 1:S67–74. doi: 10.2337/dc13-S067 PMC3537273 23264425

[14] Inker LA Schmid CH Tighiouart H Eckfeldt JH Feldman HI Greene T. Estimating Glomerular Filtration Rate From Serum Creatinine and Cystatin C. N Engl J Med (2012) 367:20–9. doi: 10.1056/NEJMoa1114248 PMC4398023 22762315

[15] Tuttle KR Bakris GL Bilous RW Chiang JL de Boer IH Goldstein-Fuchs J. Diabetic Kidney Disease: A Report From an ADA Consensus Conference. Diabetes Care (2014) 37:2864–83. doi: 10.2337/dc14-1296 PMC4170131 25249672

[16] Russo G Piscitelli P Giandalia A Viazzi F Pontremoli R Fioretto P. Atherogenic Dyslipidemia and Diabetic Nephropathy. J Nephrol (2020) 33:1001–8. doi: 10.1007/s40620-020-00739-8 32328901

[17] Chen X Yin Q Ma L Fu P. The Role of Cholesterol Homeostasis in Diabetic Kidney Disease. Curr Med Chem (2021) 28:7413–26. doi: 10.2174/0929867328666210419132807 33874866

[18] Vijay S Hamide A Senthilkumar GP Mehalingam V. Utility of Urinary Biomarkers as a Diagnostic Tool for Early Diabetic Nephropathy in Patients With Type 2 Diabetes Mellitus. Diabetes Metab Syndr (2018) 12:649–52. doi: 10.1016/j.dsx.2018.04.017 29673928

[19] Weinberg JM. Lipotoxicity. Kidney Int (2006) 70:1560–6. doi: 10.1038/sj.ki.5001834 16955100

[20] Thongnak L Pongchaidecha A Lungkaphin A. Renal Lipid Metabolism and Lipotoxicity in Diabetes. Am J Med Sci (2020) 359:84–99. doi: 10.1016/j.amjms.2019.11.004 32039770

[21] Muntner P Coresh J Smith JC Eckfeldt J Klag MJ. Plasma Lipids and Risk of Developing Renal Dysfunction: The Atherosclerosis Risk in Communities Study. Kidney Int (2000) 58:293–301. doi: 10.1046/j.1523-1755.2000.00165.x 10886574

[22] Retnakaran R Cull CA Thorne KI Adler AI Holman RR UKPDS Study Group. Risk Factors for Renal Dysfunction in Type 2 Diabetes: U.K. Prospective Diabetes Study 74. Diabetes (2006) 55:1832–9. doi: 10.2337/db05-1620 16731850

[23] Tsuruya K Yoshida H Nagata M Kitazono T Iseki K Iseki C. Impact of the Triglycerides to High-Density Lipoprotein Cholesterol Ratio on the Incidence and Progression of CKD: A Longitudinal Study in a Large Japanese Population. Am J Kidney Dis (2015) 66:972–83. doi: 10.1053/j.ajkd.2015.05.011 26145254

[24] Russo GT De Cosmo S Viazzi F Pacilli A Ceriello A Genovese S. Plasma Triglycerides and HDL-C Levels Predict the Development of Diabetic Kidney Disease in Subjects With Type 2 Diabetes: The AMD Annals Initiative. Diabetes Care (2016) 39:2278–87. doi: 10.2337/dc16-1246 27703024

[25] Tu ST Chang SJ Chen JF Tien KJ Hsiao JY Chen HC. Prevention of Diabetic Nephropathy by Tight Target Control in an Asian Population With Type 2 Diabetes Mellitus: A 4-Year Prospective Analysis. Arch Intern Med (2010) 170:155–61. doi: 10.1001/archinternmed.2009.471 20101010

[26] Lin J Hu FB Mantzoros C Curhan GC. Lipid and Inflammatory Biomarkers and Kidney Function Decline in Type 2 Diabetes. Diabetologia (2010) 53:263–7. doi: 10.1007/s00125-009-1597-z PMC2809803 19921505

[27] Öberg CM Lindström M Grubb A Christensson A. Potential Relationship Between Egfr_cystatin C_/eGFR_creatinine_ -Ratio and Glomerular Basement Membrane Thickness in Diabetic Kidney Disease. Physiol Rep (2021) 9:e14939. doi: 10.14814/phy2.14939 34254743 PMC8276256

[28] Mechanick JI Farkouh ME Newman JD Garvey WT. Cardiometabolic-Based Chronic Disease, Adiposity and Dysglycemia Drivers: JACC State-Of-the-Art Review. Am Coll Cardiol (2020) 75:525–38. doi: 10.1016/j.jacc.2019.11.044 PMC7187687 32029136

[29] Koba S Yokota Y Hirano T Ito Y Ban Y Tsunoda F. The Small, Dense LDL Phenotype and the Risk of Coronary Heart Disease: Epidemiology, Patho-Physiology and Therapeutic Aspects. Diabetes Metab (1999) 25:199–211. 10499189

[30] Kaneva AM Potolitsyna NN Bojko ER. Usefulness of the LDL-C/apo B Ratio in the Overall Evaluation of Atherogenicity of Lipid Profile. Arch Physiol Biochem (2017) 123:16–22. doi: 10.1080/13813455.2016.1195411 27347637

[31] Tani S Saito Y Anazawa T Kawamata H Furuya S Takahashi H. Low-Density Lipoprotein Cholesterol/Apolipoprotein B Ratio may be a Useful Index That Differs in Statin-Treated Patients With and Without Coronary Artery Disease: A Case Control Study. Int Heart J (2011) 52:343–7. doi: 10.1536/ihj.52.343 22188706

[32] Li G Wu HK Wu XW Cao Z Tu YC Ma Y. Small Dense Low Density Lipoprotein-Cholesterol and Cholesterol Ratios to Predict Arterial Stiffness Progression in Normotensive Subjects Over a 5-Year Period. Lipids Health Dis (2018) 17:27. doi: 10.1186/s12944-018-0671-2 29433526 PMC5810050

[33] Hirayama S Miida T. Small Dense LDL: An Emerging Risk Factor for Cardiovascular Disease. Clin Chim Acta (2012) 414:215–24. doi: 10.1016/j.cca.2012.09.010 22989852

[34] Roumeliotis S Georgianos PI Roumeliotis A Eleftheriadis T Stamou A Manolopoulos VG. Oxidized LDL Modifies the Association Between Proteinuria and Deterioration of Kidney Function in Proteinuric Diabetic Kidney Disease. Life (Basel) (2021) 11:504. doi: 10.3390/life11060504 34072583 PMC8226768

[35] Yang Y Xu P Liu Y Chen X He Y Feng J. Vascular Inflammation, Atherosclerosis, and Lipid Metabolism and the Occurrence of non-High Albuminuria Diabetic Kidney Disease: A Cross-Sectional Study. Diabetes Vasc Dis Res (2021) 18:1479164121992524. doi: 10.1177/1479164121992524 PMC8482348 33567895

[36] Hayashi T Fukui T Nakanishi N Yamamoto S Tomoyasu M Osamura A. Dapagliflozin Decreases Small Dense Low-Density Lipoprotein-Cholesterol and Increases High-Density Lipoprotein 2-Cholesterol in Patients With Type 2 Diabetes: Comparison With Sitagliptin. Cardiovasc Diabetol (2017) 16:8. doi: 10.1186/s12933-016-0491-5 28086872 PMC5237208

